# Necesidad de ajustar al IMC los valores límite de los péptidos natriuréticos para un diagnóstico más personalizado de la insuficiencia cardíaca

**DOI:** 10.1515/almed-2025-0060

**Published:** 2025-04-18

**Authors:** Damien Gruson, Sergio Bernardini, Marco Perrone

**Affiliations:** Servicio de Medicina de Laboratorio, Clínicas Universitarias St-Luc y Universidad Católica de Louvain, Bruselas, Bélgica; Red de Investigación en Endocrinología, Diabetes y Nutrición, Instituto de Investigación Experimental y Clínica, Clínicas Universitarias St-Luc y Universidad Católica de Louvain, Bruselas, Bélgica; Departamento de Medicina Experimenta, Universidad de Roma Tor Vergata, Roma, Italia; Departamento de Ciencías Clínicas y Medicina Traslacional, Universidad de Roma Tor Vergata, Roma, Italia

**Keywords:** insuficiencia cardíaca, biomarcadores, IMC, obesidad, decisión

Estimado Editor,

La insuficiencia cardíaca (IC) sigue siendo un desafío a nivel mundial, en cuyo diagnóstico y manejo desempeñan un papel fundamental los péptidos natriuréticos [1]. El empleo de dichos biomarcadores resulta esencial a la hora de distinguir entre una etiología cardíaca y no cardíaca de la disnea, así como de realizar la evaluación de riesgos en pacientes con IC y evaluar la eficacia de la terapia [1], [2].

La creciente prevalencia de la obesidad representa todo un desafío para los sistemas sanitarios, especialmente en lo relativo al manejo de la enfermedad cardiovascular. La correlación negativa entre el índice de masa corporal (IMC) y los niveles de péptidos natriuréticos dificulta la interpretación de estos biomarcadores en pacientes con obesidad, ya que estos suelen presentar niveles más bajos, lo que podría retrasar el diagnóstico de insuficiencia cardíaca, derivando así en peores resultados clínicos [3], [4]. Dicha reducción se debe a diversos mecanismos. En primer lugar, el tejido adiposo, que es abundante en los pacientes con obesidad, presenta una expresión elevada de receptores de tipo C de péptidos natriuréticos (NPR-C), lo que facilita la rápida eliminación de péptidos de la circulación, reduciendo así significativamente sus niveles [3]. Así mismo, existe evidencia de que los sujetos obesos presentan una menor liberación de péptidos miocárdicos, posiblemente debido a estados hemodinámicos alterados o a la inhibición de la síntesis de péptidos en los miocitos cardíacos mediada por adipocinas [4]. La obesidad también induce cambios en la bioquímica del sujeto, lo que podría influir en los niveles de péptidos natriuréticos. Los niveles elevados de citocinas inflamatorias podrían alterar las funciones cardíaca y renal que controlan la producción y eliminación de péptidos. La disfunción renal, que suele estar asociada a la obesidad, complica la eliminación de los péptidos natriuréticos en los riñones, que suelen estar afectados por comorbilidades comunes como la hipertensión y la diabetes [5]. Además, la expresión elevada del receptor NPR-C en la obesidad no solo incrementa la eliminación, sino que también afecta a los mecanismos de regulación hormonal, alterando el equilibrio fisiológico y contribuyendo a aumentar el ritmo de degradación de los péptidos natriuréticos [4]. Además, los factores genéticos también influyen en la síntesis, liberación o degradación de los péptidos natriuréticos, lo que repercute en su variabilidad en las poblaciones obesas [6]. El déficit observado podría tener también efectos metabólicos directos, dado que estos péptidos participan en el metabolismo lipídico y desempeñan diversas funciones en procesos como la lipólisis y la sensibilidad a la insulina, lo que agudiza la desregulación metabólica en la obesidad [4].

La complejidad de estos procesos subraya la necesidad de adoptar un enfoque más personalizado en el diagnóstico y tratamiento de patologías en pacientes obesos. Los estudios realizados por Kozhuharov y col. evidencian la necesidad de ajustar los límites de decisión de los péptidos natriuréticos en función del IMC, con el fin de mejorar la precisión diagnóstica en los pacientes con obesidad [7]. Hemos realizado estos ajustes, esto es una reducción del 33 % para un IMC de 30,0–34,9 kg/m^2^ y del 50 % para un IMC ≥35,0 kg/m, para mejorar la efectividad del NT-proBNP como biomarcador en la población obesa.

A pesar de la evidencia científica existente sobre la necesidad de ajustar los valores límite, es necesario superar primero algunas dificultades, con el fin de lograr integrarlos en la práctica clínica, tales como la inercia de los protocolos establecidos y la necesidad de consenso entre los prestadores de servicios sanitarios. Para validar los límites propuestos, es necesario realizar ensayos clínicos multicéntricos en diferentes poblaciones, entre las que se incluyan grupos de diferentes razas y etnias. Desde el punto de vista de las políticas sanitarias, las autoridades regulatorias deberían plantearse la revisión de las guías clínicas, con el fin de incorporar estos nuevos límites, y complementarlas con programas formativos para profesionales de la salud, y facilitar así la transición.

En conclusión, la creciente prevalencia de la obesidad hace necesario adaptar las estrategias diagnósticas en la insuficiencia cardíaca, especialmente en lo relativo al uso de los péptidos natriuréticos como biomarcadores. Al adoptar valores de decisión clínica ajustados al IMC para este biomarcador tan esencial, se podría mejorar la precisión diagnóstica y los resultados clínicos en el manejo de la insuficiencia cardíaca, garantizando así que todos los pacientes, independientemente de su composición corporal, reciben una asistencia médica óptima.
